# Comparing dedicated and designated models of integrating mental health into chronic disease care: study protocol for a cluster randomized controlled trial

**DOI:** 10.1186/s13063-018-2568-9

**Published:** 2018-03-16

**Authors:** Bronwyn Myers, Crick Lund, Carl Lombard, John Joska, Naomi Levitt, Christopher Butler, Susan Cleary, Tracey Naledi, Peter Milligan, Dan J. Stein, Katherine Sorsdahl

**Affiliations:** 1 Alcohol, Tobacco and Other Drug Research Unit, South African Medical Research Council, Francie van Zyl Drive, Tygerberg, Cape Town, 7505 South Africa; 2 Division of Addiction Psychiatry, Psychiatry and Mental Health, University of Cape Town, Cape Town, South Africa; 3 Alan J Flisher Centre for Public Mental Health, Department of Psychiatry and Mental Health, University of Cape Town, Cape Town, South Africa; 4 Centre for Global Mental Health, Institute of Psychiatry, Psychology and Neuroscience, King’s College London, London, UK; 5 Biostatistics Unit, South African Medical Research Council, Francie van Zyl Drive, Tygerberg, Cape Town, 7505 South Africa; 6 HIV and Mental Health Research Unit, Division of Neuropsychiatry, Department of Psychiatry and Mental Health, University of Cape Town, Cape Town, South Africa; 7 Division for Diabetes and Endocrinology, Department of Medicine, University of Cape Town, Cape Town, South Africa; 8 Nuffield Department of Primary Care Health Services, Oxford University, Oxford, UK; 9 Health Economics Unit, School of Public Health and Family Medicine, University of Cape Town, Cape Town, South Africa; 10 Western Cape Department of Health, 8 Riebeeck Street, Cape Town, South Africa; 11 Department of Psychiatry and Mental Health, University of Cape Town, Cape Town, South Africa; 12 Western Cape Department of Health, Valkenberg Hospital, Cape Town, South Africa; 13 Department of Psychiatry and Mental Health, University of Cape Town, Cape Town, South Africa; 14 Unit on Anxiety and Stress Disorders, South African Medical Research Council, Cape Town, South Africa; 15 Alan J Flisher Centre for Public Mental Health, Department of Psychiatry and Mental Health, University of Cape Town, Cape Town, South Africa

**Keywords:** Common mental disorders, Chronic disease care, Integrated treatment, South Africa

## Abstract

**Background:**

In low- and middle-income countries (LMIC), it is uncertain whether a “dedicated” approach to integrating mental health care (wherein a community health worker (CHW) has the sole responsibility of delivering mental health care) or a “designated” approach (wherein a CHW provides this service in addition to usual responsibilities) is most effective and cost-effective. This study aims to compare the effectiveness and cost-effectiveness of these two models of service integration relative to treatment as usual (TAU) for improving mental health and chronic disease outcomes among patients with HIV or diabetes.

**Methods/Design:**

This is a cluster randomised trial. We will randomise 24 primary health care facilities in the Western Cape Province of South Africa to one of three study arms. Within each cluster, we will recruit 25 patients from HIV and 25 from diabetes services for a total sample of 1200 participants. Eligible patients will be aged 18 years or older, take medication for HIV or diabetes, and screen positive on the Alcohol Use Disorder Identification Test for hazardous/harmful alcohol use or depression on the Centre for Epidemiology Scale on Depression. Participants recruited in clinics assigned to the designated or dedicated approach will receive three sessions of motivational interviewing and problem-solving therapy, while those recruited at TAU-assigned clinics will be referred for further assessment. Participants will complete an interviewer-administered questionnaire at baseline, and at 6 and 12 months post-enrolment to assess change in self-reported outcomes. At these end points, we will test HIV RNA viral load for participants with HIV and HbA1c levels for participants with diabetes. Primary outcomes are reductions in self-reported hazardous/harmful alcohol use and risk of depression. Secondary outcomes are improvements in adherence to chronic disease treatment, biomarkers of chronic disease outcomes, and health-related quality of life. Mixed-effect linear regression models will model the effect of the interventions on primary and secondary outcomes. The cost-effectiveness of each approach will be assessed using incremental cost-effectiveness ratios.

**Discussion:**

Study findings will guide decision-making around how best to integrate mental health counselling into chronic disease care in a LMIC setting.

**Trial registration:**

Pan African Clinical Trials Registry, Trial registration number: ACTR201610001825403. Registered 17 October 2016.

**Electronic supplementary material:**

The online version of this article (10.1186/s13063-018-2568-9) contains supplementary material, which is available to authorized users.

## Background

Like many low- and middle- income countries (LMICs) [1, 2], South Africa has a high burden of mental disorders, with an estimated 30% of the adult population meeting diagnostic criteria for a mental disorder at some point in their life [3]. In South Africa, a substantial treatment gap exists for adults with common mental disorders (CMDs), that includes mood, anxiety and alcohol use disorders: approximately 75% of those affected never access mental health treatment [4]. Health providers can be reluctant to screen patients for possible CMDs because there are limited options for treatment in the public health care system and they usually work under considerable pressure [5–7]. Publically funded mental health treatment focuses on managing severe mental disorders within specialist psychiatric hospitals, with limited services being available in primary health care (PHC) services [8]. In addition, the mental health services that are available in PHC clinics generally focus on medication provision, with mental health counselling rarely provided [7–9].

In South Africa, there is a public health imperative to address this treatment gap, given evidence of the high comorbidity of CMDs with other health conditions [10]. Untreated mental and neurological disorders are the third largest direct contributor to the national burden of disease and also contribute to the national burden of disease associated with chronic communicable diseases (such as HIV) and non-communicable disease (NCDs), such as diabetes [10]. For example, South African studies have shown high levels of CMD and NCD multi-morbidity among the general population [11, 12] and patients attending PHC facilities [13]. Evidence suggests that maladaptive coping and problem-solving skills play a pivotal role in both CMDs and chronic diseases [14–16]. Difficulties in accepting and disclosing a chronic disease diagnosis may lead to depression [17]. Often alcohol is a (maladaptive) way of coping with having a chronic disease [15]. As untreated depression and alcohol use disorders are associated with sub-optimal adherence to chronic disease treatment, and consequently increased risk of treatment failure and negative treatment outcomes [18, 19], ensuring access to treatment for these CMDs is an essential component of an effective health system response to chronic disease.

Integrating mental health care into chronic disease services offered in PHC clinics has been widely proposed as a means of reducing the mental health treatment gap and improving chronic disease outcomes in LMICs [20–23]. Although South African health policies call for the integration of mental health and chronic disease services [24], shortages of mental health providers in the country have hampered integrated service delivery [21, 25, 26]. To overcome this barrier, South Africa’s mental health care policy framework [27] has embraced task-shifting of basic mental health counselling from specialty mental health providers to community health workers (CHWs). Task-shifting basic health services to CHWs has emerged as a strategy for addressing shortfalls in human resources impeding the delivery of health programmes in LMICs [28]. There is some evidence to suggest that brief, structured counselling interventions, when task-shifted to trained CHWs, may improve patients’ mental health outcomes [29]. For example, three to four sessions of motivational interviewing (MI) integrated with problem-solving therapy (PST), two of the recommended interventions from the World Health Organization’s programme to reduce the mental health treatment gap [30], improved depression and alcohol outcomes among PHC patients in South Africa [31–34]. There is good evidence that combining MI and cognitive-behavioural treatments (such as PST) leads to improved outcomes for patients with depression [35, 36], alcohol and other substance use disorders [34], and patients with comorbid alcohol use disorders and depression [37].

Despite this evidence, uncertainty about how to configure services and resources within the PHC system to allow for the effective integration of mental health counselling has hindered the implementation of integrated mental health and chronic disease services [25, 38]. On one hand, some have shown that basic mental health counselling can be effectively task-shifted to CHWs already working in PHC settings [29, 39, 40], suggesting that resources available in the PHC system can be reconfigured to enable the delivery of new programmes. In contrast, others have argued that CHWs already in the PHC system are overburdened with multiple tasks and therefore unlikely to prioritise the provision of mental health counselling (and may lack the skills and training to be effective), noting that additional human resources are needed for effective integration of mental health and chronic disease services [41–43]. However, health care systems are unlikely to finance additional resources without evidence of the cost-effectiveness of this investment relative to other approaches to service integration [44]. To the best of our knowledge, there has been no research comparing the relative effectiveness and cost-effectiveness of these approaches [29]. Addressing this knowledge gap is critical for guiding decision-making around how to proceed with integrating mental health into chronic disease care within South Africa’s PHC system.

### Trial objectives

The goal of this trial is to identify an effective and cost-effective approach for integrating mental health counselling into chronic disease care in the Western Cape province of South Africa. The primary objective is to compare the effectiveness of a designated approach (where an existing CHW is tasked with providing mental health counselling in addition to their usual responsibilities), relative to a dedicated approach (where a CHW solely responsible for mental health counselling is added to the chronic disease care team), relative to treatment as usual (TAU) for reducing depression and hazardous/harmful alcohol use.

Secondary objectives include comparing the effectiveness of the interventions for improving chronic disease outcomes (HIV1 RNA viral load for HIV and HbA1c for diabetes), adherence to chronic disease treatment, and health-related quality of life (HrQoL) and exploring potential mediators and moderators of the effects of the interventions on primary outcomes. In addition, we will compare the cost-effectiveness of each intervention alternative from a societal perspective.

## Methods

This manuscript is in accordance with the Standard Protocol Items: Recommended items to address in a clinical trial protocol and related documents (SPIRIT) guidelines. See Additional file 1 for the Standard Protocol Items: Recommendations for clinical trial protocol (SPIRIT) checklist.

### Trial design

**Fig. 1:**
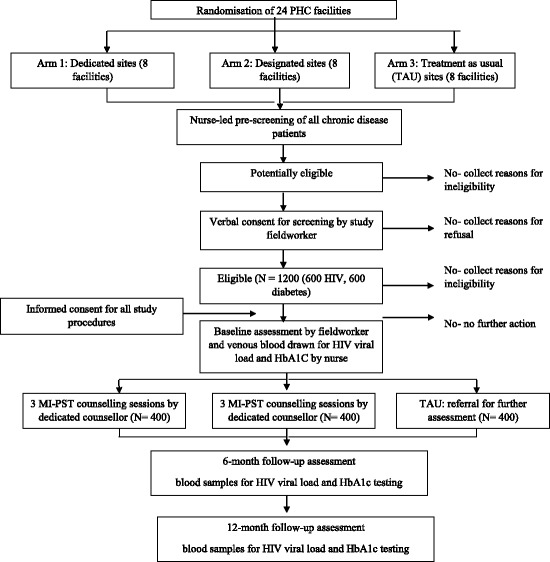
Consort diagram of the study design

Project Mind is a three-arm, cluster randomised controlled trial, with the clusters being Western Cape Department of Health (WCDOH) PHC clinics that offer HIV and diabetes treatment and care (see Consort diagram in Fig. 1). The intervention will influence the staff delivering HIV and diabetes services within PHC clinics, thus randomisation is at the level of the PHC clinic to avoid contamination.

### Study sites: selection and randomisation

The trial will be conducted at 24 co-located HIV and diabetes treatment clinics in the Western Cape. For these clinics to be included in the trial, they needed to treat large enough numbers of patients to facilitate patient recruitment and be co-located within PHC facilities. For the most part, HIV and diabetes services are provided by separate, vertically organised clinics co-located within the same PHC facility; this study requirement therefore is unlikely to introduce a selection bias. The WCDOH purposively selected PHC facilities to participate in the trial. Selected facilities vary in size and the extent to which comprehensive health services are provided in order to be broadly representative of the mix of PHC facilities available in the province. Facilities are located in most of the health districts in the province, with 15 being urban and 9 being rural sites. This urban: rural ratio is reflective of the provincial distribution of PHC clinics.

An independent statistician will conduct randomisation after all PHC facilities selected to participate in the trial formally agree to participate. Randomisation will be based on randomly generated numbers (computer based), stratified by urban-rural status. Within each stratum, facilities will be allocated to the three study arms with equal probability (1:1:1 ratio). Specifically, facilities will be assigned a random number between 0 and 1 using the rand() function in Excel. Then these facilities will be sorted in ascending order based on the random numbers, and the first third of facilities will be assigned to the dedicated arm, the second to the designated arm and the last third to the TAU arm.

It is not possible to blind investigators involved in the delivery of the intervention to group allocation. In addition, due to the nature of the intervention, it is not possible to blind the facilities to group allocation. Investigators will be blind to the sequence generation, and all allocations will be concealed until the facility is assigned to a study arm. Further, CHWs delivering the intervention and outcome assessors administering patient questionnaires will function independently of each other: CHWs will not conduct any assessments, ensuring that these assessments remain independent from the counselling sessions and assessors collecting outcome data remain blind to the content of the counselling sessions.

### Patient recruitment and study procedures

Eligible patients will be at least 18 years of age, take antiretroviral therapy (ART) for HIV or medication for diabetes, screen for hazardous/harmful alcohol use (Alcohol Use Disorders Identification Test (AUDIT) score ≥ 8) or depression (Center for Epidemiology Scale on Depression (CES-D) score ≥ 16), not be currently receiving treatment for a mental health condition, and not be participating in another study.

**Fig. 2:**
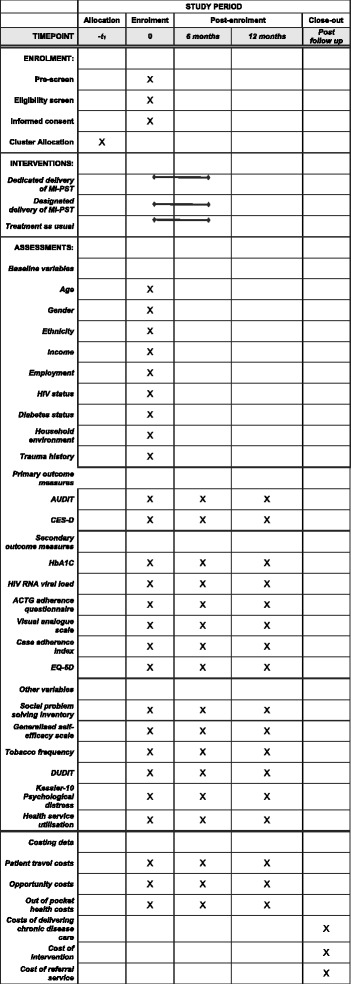
Standard Protocol Items: Recommendations for Clinical Trials (SPIRIT) Figure: schedule of enrolment, interventions and data collection

During the study period, all patients presenting for HIV or diabetes care will be asked about their use of alcohol in the past year and recent low mood by their health care provider. Patients who respond positively to one of these two questions will be referred to an assessor for study eligibility screening. After explaining the purpose of the study, the assessor will request verbal consent to screen the patient for possible study inclusion. If a patient is eligible and interested in participating, the assessor will make an appointment for the enrolment visit. At this visit, the assessor will re-screen the participant for study eligibility prior to obtaining informed consent to participate in the trial. Thereafter the assessor will administer the baseline assessment in either English, Afrikaans or isiXhosa, the three official languages of the Western Cape province. This computer-assisted personal interview collects socio-demographic information as well as information on HIV disease, adherence and treatment; diabetes disease, adherence and treatment; CMDs; patterns of health service use and associated patient costs; self-efficacy; readiness to change; problem-solving styles; social support and HrQoL (see Fig. 2 for a description of the measures). Next, a registered nurse will draw up to 10 ml of venous blood to assess the participants’ HIV viral load and HbA1c levels, as appropriate. The blood samples will be sent to a local accredited laboratory for analysis. The assessor will then schedule counselling appointments for participants recruited from facilities assigned to the intervention arms. Ideally, the first session will take place within 3 days of the enrolment visit, with the remaining two sessions scheduled 5 days apart. Participants will have 6 weeks from enrolment to complete these three sessions.

Irrespective of study arm, all participants will be asked to return to the facility at 6 and 12 months’ post-enrolment to complete a follow-up assessment. During these follow-up visits, the assessor will re-administer the baseline assessment and the nurse will draw blood samples for repeated HIV viral load and HbA1c testing (see Fig. 2). Participants will have 30 days from their scheduled appointment to complete these follow-up assessments before timing out of that particular appointment. Transport costs associated with attending study or counselling appointments will be reimbursed. Participants will receive grocery vouchers for completing the baseline and follow-up assessments.

### Interventions

#### Control: TAU arm

Participants recruited from facilities assigned to the control arm will receive the standard package of care when a health provider suspects a patient may have a CMD. This standard of care involves asking patients about their mood, stressors and their use of alcohol when patients present for HIV or diabetes care. If a patient discloses difficulties in these areas, the patient may be given lifestyle advice and provided with a referral to an on-or off-site mental health nurse or social worker for further assessment [45]. We have previously found that the implementation of this standard of care varies considerably among facilities. Consequently, we will provide all participants recruited in TAU facilities with a resource guide that contains mental health and substance abuse services within their health sub-district and a referral to a service provider of their choice.

#### Interventions: “Dedicated” and “Designated” arms

Participants recruited from facilities in the dedicated and the designated arms will receive three sessions of MI-PST counselling and be referred for further mental health treatment, if needed. Each session is about 60 minutes in duration. In addition, participants will be offered a telephonic booster session 8 weeks after study enrolment. Previous studies have shown that three to four sessions of MI-PST are feasible to implement in PHC settings, acceptable to CHWs and service users, (with attendance declining after three sessions), and impact positively on mental health outcomes [31–33]. The underlying theory and content of this structured counselling approach, and evidence of its efficacy for reducing hazardous/harmful alcohol use and symptoms of depression have been described previously [31–34]. The goals of MI-PST are to motivate participants to change their risk behaviours and to improve their problem-solving skills so they cope better with stress and life problems that are risk factors for CMDs and poor adherence to chronic disease treatment [13–15]. During the sessions, the counsellor and the participant collaborate to identify problems within the participant’s life, and explore one or more of these problems while the counsellor teaches the participant a structured PST approach to resolving these problems. Each session functions iteratively to build readiness to change and problem-solving skills. All sessions have a motivational component, an education component (in which participants are taught problem-solving skills and how to apply them), and includes opportunities to apply newly learned skills through exercises and take home activities. More specifically, participants are taught steps for addressing problems that are important and can be solved; strategies for dealing with negative and intrusive worries that are unimportant; and steps for coping with problems that are important but cannot be solved. These activities are contained in a patient handbook that also summarises the content of the sessions.

Therefore, it is intended that participants in both intervention arms will receive the same counselling programme, but that the job scope of the person delivering the counselling will differ. In facilities assigned to the dedicated arm, one CHW will be added to each facility (eight in total). This CHW will be tasked with providing the counselling programme to patients with HIV and those with diabetes, but will have no other formal chronic disease-related duties. If they have spare capacity, they may assist with administrative and other tasks for the chronic disease care team, but their priority will be the delivery of project MIND’s mental health counselling package. Although these CHWs will be employed as part of the study, their salaries and conditions of employment will match those of the CHWs in the designated arm. In the designated arm, we will identify eight CHW (one per facility) already employed as facility-based counsellors who will be designated to provide the counselling programme to patients with HIV and those with diabetes in addition to their usual chronic disease-related counselling responsibilities. Both dedicated and designated CHWs will be required to complete a time and motion tool that captures their activities and the time spent on each activity on a daily basis. Activities may include mental health counselling, administrative tasks, participation in supervision or training, and other chronic disease activities, which they will be required to specify. This will allow for an exact understanding of their daily activities to be developed.

#### Counsellor training and supervision

Regardless of arm, each CHW will initially receive the equivalent of 40 h of training in understanding CMDs, screening for hazardous/harmful alcohol use and depression, principles of basic counselling, the MI and PST programme, referral pathways, and responding to distressed participants. Prior to the start of training, CHWs will complete an assessment of their knowledge, attitudes, beliefs and practices around counselling for CMDs, and these will be assessed after completion of training to evaluate the impact of training. Role-plays and observations will assess counsellors’ proficiency in delivering the counselling programme. Each counsellor will receive refresher training between 2 and 3 months after the start of the study. A psychological counsellor will conduct regular face-to-face and virtual supervision with the counsellors in order to review participants’ progress, provide feedback on how the quality of counselling can be improved, and allow for the prevision of additional training if required. The number of hours spent on training and supervision per individual CHW will be recorded in a training and supervision log.

#### Treatment fidelity and monitoring

For pragmatic reasons, we will not assess the fidelity with which standard care is delivered, although we will collect information on whether participants were referred to mental health services and we will ask participants about the use of these services at follow-up. For the dedicated and designated arms, we will assess treatment fidelity using the five steps recommended by the Behavioral Change Consortium’s treatment fidelity framework [46]. To evaluate the impact of training, we will assess changes in CHWs’ knowledge, attitudes, beliefs and practices around CMD counselling after completion of training. Second, we will audio-record counselling sessions to measure treatment differentiation (whether the CHWs delivered the intended programme and not another programme), competency (whether the CHWs maintained the counselling skills learned during training), and adherence (delivery of the intervention components as intended) [43]. We will select a random sample of audio-recorded counselling sessions to review for each counsellor. Third, we will assess whether the participant understood and retained the content of treatment by enquiring whether participants completed their take home activities and whether they had any difficulty with these assignments. Fourth, we will analyse the audiotapes of the counselling sessions to assess the degree of counselling enactment, or the extent to which the participant transferred the skills and strategies learned during counselling to real-life problems [43]. We will report on the training of the CHWs in the main trial publication, there will be a separate publication on treatment fidelity.

### Study measures

The schedule of data collection is shown in Fig. 2, the SPIRIT Figure.

#### Primary outcome measures

All primary outcomes are at the level of the individual patient and will be collected at baseline and the two follow-up assessments.

*Hazardous/harmful alcohol use* as measured by the 12-item Alcohol Use Disorders Identification Test (AUDIT) [47]. The AUDIT has been validated for use among South African patients utilising health care facilities [48].

*Depressive symptomatology* as measured by the Center for Epidemiologic Studies Depression Scale (CES-D), a 20-item scale that measures depressive feelings and behaviours during the past week [49] and has been used to measure change in symptoms of depression among PHC patients [31, 34].

#### Secondary outcome measures

##### Biomarkers of chronic disease treatment outcomes

HbA1c levels (for diabetes) and HIV-1 RNA viral load levels (for HIV) will measure change in risk for chronic disease treatment failure. Biologic measures will be recorded as continuous variables and dichotomized as normal or abnormal using standard cut-offs (> = 7 for HbA1c and > = 1000 copies/ul for viral load).

##### Adherence to chronic disease treatment

Three measures will assess adherence to HIV and diabetes treatment, respectively. The AIDS Clinical Trials Group (ACTG) adherence questionnaire assesses patients’ current medications, dosing schedule, and medication doses missed over the past four days [50]. The Visual Analog Scale (VAS) assesses general levels of adherence over a 30-day timeframe [51]. For the ACTG and VAS, adherence levels will be examined as continuous variables and dichotomised using standard cut-off scores for adherence (e.g. > = 90%). The third measure is the CASE Adherence Index [52]. Questions on barriers to adherence are also included.

##### Health-related quality of life (HrQoL)

The EuroQol 5 (EQ-5D) will assess HrQoL. The EQ-5D generates a single index that measures how much someone’s life could be extended and improved [53]. From this index, quality-adjusted life years (QALY) will be estimated.

#### Possible mediators and moderators of outcomes

We will collect information on a variety of variables thought to potentially mediate or moderate response to the counselling intervention. The 25-item Social Problem-Solving Inventory Revised-Short Form will be used to assess problem-solving styles. This has been adapted for use among South African populations [54]. The MOS Social Support survey will assess nature and extent of social support [55]. We will also use the 10-item Generalized Self-efficacy Scale to assess participants’ confidence in their ability to solve and cope with a range of problems [56] and the Trauma History Questionnaire will examine lifetime trauma exposure [57]. We will assess other risk behaviours that may impact on study outcomes. We will examine frequency, quantity and chronicity of tobacco use. In addition, the 11-item Drug Use Disorders Identification Test will assess harmful patterns of illicit drug use and dependence [58]. The 10-item Kessler psychological distress scale will examine extent of psychological distress [59].

#### Economic/costing measures

Full economic costs will be assessed from a societal (i.e. provider and patient) perspective within each of the three study arms. For the provider perspective, costs include the costs of the dedicated/designated approach, the costs of chronic disease (HIV/diabetes) care, and the costs of a range of referral services (e.g. referral for TB treatment or inpatient care). While TAU does not include any counselling costs, our inclusion of chronic disease and referral costs for each of the three arms ensures that any potential changes to a broader range of costs is captured. Provider costs will be estimated using a combination of ingredients and step-down methods. A time and motion study will capture the time spent by dedicated or designated CHWs on their daily activities. This will enable an assessment of excess capacity (hypothesized to occur in the dedicated CHWs) and economies of scope (hypothesized to occur in the designated CHWs). Ultimately, a provider cost per patient year will be computed for each study arm/chronic disease.

In the patient perspective, costs include travel costs, the opportunity costs of travel and waiting times, productivity gains or losses and out-of-pocket payments (such as food purchased during a clinic visit, user fees for a private hospital stay, and the costs of additional over-the-counter medicines). Using patient questionnaires, these data will be collected for all of the health services utilized by the patient during the month preceding the interview for the baseline and follow-up assessments (including additional mental health services). We will extrapolate these data in order to calculate a patient cost per patient year for each study arm/chronic disease. In addition, we will collect a range of socioeconomic and demographic data to enable an assessment of patient costs and catastrophic expenditure by socioeconomic status.

### Sample size considerations

This trial is powered to detect reductions in hazardous/harmful alcohol use and risk of depression at 12-month follow-up. The total sample size required to assess these outcomes is 1200 participants. Sample size calculations are based on a cluster randomised design with two active arms and a control arm. The sample size is based on separate analyses of diabetes and HIV clinic populations, showing a difference between the active arms using two-sided tests at α = 0.05 and 90% power. The statistical comparison will be the mean scores of the CES-D and AUDIT at 12-month follow-up. Guided by international and local cluster trials of depression and alcohol use among primary care patients which found ICCs ranging between 0.025 and 0.040 [60, 61], we set the intra-class correlation at 0.030, which is reasonable for outcomes in patients from PHC facilities. Given findings from previous MI [62, 63] and MI-PST [34] interventions, we expect to find a three-unit difference in AUDIT scores at follow-up between the active arms (considered clinically significant) and a between-person standard deviation of 6.5. For this specification with 20 patients per cluster, eight clinics per arm are needed (24 in total). Guided by findings of PST interventions for depression [34, 64], we anticipate a five=unit difference in CES-D scores between the active arms (considered clinically significant) and a between-person standard deviation of 10 units. For this specification, with 20 participants per cluster, seven clinics per arm are needed (21 in total). We will inflate the cluster size of 20 participants to 25 participants per cluster to cater for a worst-case scenario of a 20% attrition rate. Consequently, a sample size of eight clinics per arm (24 HIV and 24 diabetes services in total), with a cluster size of 25 participants (600 unique participants from HIV and 600 unique participants from diabetes clinics) will meet the inference requirements of the trial. During our pilot phase, we recruited participants with depression and those with hazardous/harmful alcohol use in a 2:1 ratio. To ensure we have sufficient power to detect change in alcohol outcomes during the trial phase, we will examine the number of recruited participants who are eligible based on their alcohol use once we have completed recruitment in each site. If necessary, we will augment the sample by recruiting additional participants with hazardous/harmful alcohol use until the required number of 25 is obtained.

### Data analysis

#### Primary analyses

With 24 clusters, individual-level analyses of trial outcomes are possible. Mixed-effect linear regression models will be used with clusters and participants specified as random effects to take account of the clustering at the facility level and the repeated measures within participants. We will create separate models for alcohol and depression. The analysis will be intention-to-treat. For cluster RCTs, the dropout of clusters is rare. We will consider using multiple imputations to account for missing responses of participants at follow-up in order to reflect the true overall uncertainty of the estimated effect sizes. The two primary outcomes will be ascertained at three time points for each participant. This repeated measure feature will be used to estimate the intervention effects and 95% confidence intervals for the two primary outcomes at 12 months for three contrasts (the contrast between the Dedicated arm and TAU arm, the contrast between the Designated arm and TAU, and the contrast between the two active arms (Dedicated versus Designated), which will serve as the main comparison). Our primary analysis of outcomes will include adjustment for stratification variables. No adjustment for multiplicity will be made since the trial outcome will be determined by the overall significance of the group (intervention) effect. A similar approach will be adopted to estimate the intervention effects and 95% confidence intervals for the secondary outcomes.

#### Cost-effectiveness analysis

Decision analytical models will be developed to assess intervention costs and outcomes per patient year, from the societal, provider and patient perspective. Discounting will be considered when appropriate. Incremental cost-effectiveness ratios will be used to compare the relative differences between TAU and each active arm. From the provider perspective and within each arm, the cost per patient year will be the product of the utilization of different service units (for example counselling sessions or hospital services) and the unit cost per service. This approach enables uncertainty to be assessed using probabilistic sensitivity analysis on utilization elements and caters for the inherently skewed nature of cost data. From the patient perspective, data collected during patient assessments at baseline and each follow-up will be analyzed using previously established methods [65]. While cost-effectiveness ratios will be computed for each primary outcome measure, QALY measures will allow cost-effectiveness results to be compared with alternative interventions and for allocative efficiency to be assessed.

#### Moderator and mediator analyses

Moderator analysis will be performed by including the moderator variable as an additional fixed factor or covariate in the regression models, and examining interaction effects. The following moderator analyses are planned: (a) educational level (completed high school), (b) readiness to change, (c) baseline AUDIT score (8–12 versus > = 13), (d) tobacco use (yes/no), (e) illicit drug use (yes/no) and (f) HrQoL subgroups based on the EQ-5D scale. Participants who are less ready to change [66] and with more severe problems [67] (as reflected in higher baseline AUDIT scores, co-occurring tobacco use and illicit drug use and lower QoL) could respond differently to the counselling programme compared to participants who are ready to change and have less severe problems, respectively. We will also use multi-level structural equation modelling to test a multi-mediation model that assesses whether problem-solving skills, self-efficacy and social support mediate the effect of the intervention on outcomes and the extent to which individual variables mediate the effect conditional on the presence of other mediators in the model.

### Ethical considerations

The South African Medical Research Council (EC 004–2/2015), the University of Cape Town (089/2015), and Oxford University (OxTREC 2–17) provided ethical approval for this study. The Western Cape Department of Health also approved this study (WC2016_RP6_9). The trial is registered with the Pan African Clinical Trials Registry (PACTR201610001825403). Informed consent will be obtained from all potential individual participants prior to enrolment. Prospective participants will be informed of all foreseeable risks of study involvement, that participation is voluntary and will not affect their clinical care, and that they may withdraw their consent without this affecting their medical care. The consent forms are available in English, Afrikaans and isiXhosa, the main languages spoken in the region.

To ensure confidentiality, a unique participant identification number will link the various study forms. We will collect data using an electronic data collection platform that will transmit data in real time to a central database, enhancing data safety. The study database will be password-protected following standard password safety procedures. The data manager will review these data daily for quality assurance and quality control purposes. All data not stored electronically will be stored in double-locked filing cabinets in designated locked offices. Forms with personal identifying information will be stored separately from case report forms. Study records will not be available to participants’ health care providers. We will however ask participants’ consent to share their HbA1c and HIV RNA viral load test results with their providers as these results may improve the clinical care they receive.

The main risk associated with this study, shared with other psychotherapy interventions, is initial worsening of symptoms and risk of suicide arising from issues uncovered during counselling [68]. To minimize this potential harm, we will train staff to screen all participants who report or display signs of distress for risk of suicide (using the Columbia Suicide Severity rating scale [69]) and to provide referrals (based on severity of risk) to specialised mental health services. There are also potential benefits to participation. All participants will benefit from screening for CMDs and referral to mental health care as appropriate. All participants will obtain more regular chronic disease monitoring (biannual versus annual) which may lead to better disease management. We anticipate that participants in the intervention arms will improve their mental health, adhere better to chronic disease care and reduce their risk of chronic disease treatment failure. Finally, on completion of the trial, all participating clinics (regardless of allocation) will be trained in the integrated approach found to be most effective, thus creating opportunities for improved clinical care for patients with chronic diseases.

### Trial management

A trial steering committee will be convened to provide overall supervision of the trial, ensure its conduct is in accordance with the principles of relevant regulations, and monitor data and trial safety. Within the trial steering committee, a smaller data safety monitoring board will monitor adverse and serious adverse events related and unrelated to the trial. In addition, we will create a stakeholder advisory group (SAG) comprising representatives from the WCDOH, health care providers, CHWs, non-governmental organisations and mental health and chronic disease service users. The SAG functions to ensure the trial is responsive to the needs of the health system, its partners and service constituency and to consider how findings from the trial can guide the implementation of mental health counselling in PHC facilities.

## Discussion

In recent years, there have been several global mental health initiatives to identify and strengthen the core elements of health systems required for integrating mental health counselling into PHC services [21, 25] and on implementing task-shifted mental health interventions into primary care services [33]. Some of these initiatives have advocated for additional staffing resources to enable the delivery of integrated care, while others have used staffing resources already present in the system to deliver these additional services. Yet, uncertainty about which of these approaches to service integration is the most effective and cost-effective has hampered the scale-up of mental health counselling [25, 33]. The proposed study will respond to this question by comparing the relative benefits of these two common approaches to mental health service integration on both mental and physical health outcomes. These findings will advance scientific knowledge in the field of mental health service implementation by identifying a model of mental health service integration that is effective and cost-effective.

This study has several strengths. The inclusion of chronic disease biomarkers and patient-level economic cost data is novel and allows for the objective demonstration of the benefits of treating CMDs for chronic disease outcomes as well as potential productivity gains. Another strength is the generalizability of findings and thus potential for high impact. First, we will be recruiting participants across multiple HIV and diabetes clinics in the Western Cape. As we are working across multiple clusters located in both rural and urban communities, findings are likely to be generalizable to other HIV and diabetes services in the province. Second, chronic disease services in the Western Cape are similar to chronic disease services in other parts of South Africa: they are busy, under-resourced, and a significant proportion of patients have co-occurring mental disorders [25]. Third, this study examines mental health counselling integration in a chronic communicable and NCD service provided by vertically distinct services co-located within PHC clinics. Should either approach to service integration prove more effective and cost- effective for both HIV and diabetes service settings, we would have identified an approach to mental health service integration with potential applicability across a range of chronic disease services. The likelihood of this study influencing health services is good as senior officials from the WCDOH are part of the investigative team and SAG, enhancing the likelihood that findings from this study will be able to influence the roll out of integrated mental health care services [70]. Further, as the generalizability of outcomes to more than one type of chronic disease and more than one region may influence the implementation of integrated mental health care, findings from this study have the potential to strengthen and expand access to mental health treatment for chronic disease patients.

Fourth, findings from this study may be generalizable to other similar LMICs. South Africa and other LMICs are experiencing an epidemiological transition from a disease burden dominated by communicable diseases to one characterised by both chronic communicable and NCDs [71]. As South Africa faces a quadruple burden of communicable diseases, NCDs, injury and mental disorders [1], it provides an ideal laboratory for health system interventions that are potentially useful for other LMICs experiencing similar epidemiological transitions. With this epidemiological transition in mind, the Sustainable Development Goals initiative has challenged countries to achieve a 30% reduction in premature deaths due to NCDs by 2030 [72]. In order to achieve this goal of improving NCD treatment outcomes, the delivery of NCD services within LMICs requires strengthening. Similar to other LMICs, South Africa’s PHC system has focused primarily on responding to the burden associated with communicable diseases, with NCD services being less resourced and developed [73]. As project MIND will generate information on the relationship between mental health counselling, treatment adherence, and clinical outcomes of NCD treatment outcomes, it has the potential to guide efforts to strengthen NCD services both locally and in other LMICs.

Despite these strengths, we anticipate some challenges to trial implementation. Recruitment of individual participants may pose challenges given the reliance on facility staff for the initial identification of suitable patients to approach for screening. To address this potential challenge to recruitment, we have developed a PHC facility engagement strategy that includes regular meetings with facility management, implementation readiness workshops to build a shared commitment to the study, joint trouble-shooting of challenges, and regular feedback of study progress at facility meetings. Second, retention of participants in a three-session intervention and through the lifespan of the study may pose some challenges. We have a comprehensive strategy to limit attrition (including reimbursement of transport costs) that has been used in other studies where we have obtained more than 80% follow-up rates [74]. Third, there are other ongoing initiatives to integrate and strengthen PHC services in the province; these other initiatives and contextual differences between facilities may affect study findings. We will collect contextual information and information on other service improvement initiatives regularly from each clinic in order to account for any additional bias. Another potential challenge relates to the quality of counselling provided by CHWs given that CHWs may have low levels of mental health literacy. Although the quality of counselling has a significant influence on the proposed outcome, in order to enhance the quality of care in both of the intervention arms, we plan to monitor the quality and idelity of counselling closely. We also plan to interview participants about their experiences of counselling. Through monitoring and participant feedback, we will learn about the amount of training and supervision needed to develop mental health care competencies among CHWs. This information is likely to be relevant for the implementation of other health programmes involving CHWs.

In summary, this study has the potential to fill an important knowledge gap regarding effective and cost-effective approaches to integrating mental health counselling into chronic disease care. Evidence generated by this study will be of direct relevance to current efforts to reform the public health system in SA and other LMICs where there is a focus on expanding access to mental health care [23–25]. To ensure that study findings are used to expand access to mental health counselling, we are planning to provide all participating clusters with training on the service integration model found to be most effective and cost-effective.

## Trial status

Enrolment of PHC clusters began on 19 April and individual participants began on 17 May 2017. Recruitment is ongoing and is expected to run through December 2018.

## Additional file

Additional file 1: SPIRIT 2013 Checklist: recommended items to address in a clinical trial protocol and related documents. (DOC 120 kb)

## Abbreviations

ART: Antiretroviral therapy
AUDIT: Alcohol Use Disorders Identification Test
CES-D: Center for Epidemiology Scale on Depression
CHW: Community health worker
CMD: Common mental disorder
HrQoL: Health-related quality of life
LMIC: Low- and middle-income country
MI: Motivational interviewing
NCD: Non-communicable disease
PHC: Primary health care
PST: Problem-solving therapy
QALY: Quality-adjusted life year
SAG: Stakeholder Advisory Group
TAU: Treatment as usual
WCDOH: Western Cape Department of Health
